# The Oviposition Site Selection and Photoperiod Regulation of *Megalurothrips usitatus* (Thysanoptera: Thripidae) in Different Cowpea Plant Parts

**DOI:** 10.3390/insects17080806

**Published:** 2026-08-04

**Authors:** Jiachen Huang, Zikai Han, Wangpeng Shi

**Affiliations:** 1 College of Plant Protection, China Agricultural University, Beijing 100193, China; eddyh2004@163.com; 2 College of Grassland Science and Technology, China Agricultural University, Beijing 100193, China; x3454189098@163.com; 3 MOA Key Laboratory of Pest Monitoring and Green Management, Department of Entomology, College of Plant Protection, China Agricultural University, Beijing 100193, China

**Keywords:** bean flower thrips, insect behavior, light-dark cycle, hatching rate, plant parts selection

## Abstract

*Megalurothrips usitatus* (Thysanoptera: Thripidae) is a key destructive pest of cowpea, resulting in considerable economic losses. Understanding *M. usitatus*’s oviposition preference in various plant parts under distinct photoperiod treatments is important for its success in pest control. We placed mated females with cowpea stems, leaves and pods in rearing boxes. After 24 h, eggs were counted after basic fuchsin staining. Hatched nymphs were recorded over 7 days. Morphology and histology were examined by microscopy and scanning electron microscopy. A statistical comparison on the number of egg-laying and hatching nymphs was conducted in different cowpea parts under photoperiod (L:D = 18:6) and continuous darkness (L:D = 0:24). Under photoperiod (L:D = 18:6), oviposition preference ranked as follows: pods (61.7%) > leaves (29.4%) > stems (8.9%). Nymph numbers followed the same order (79.5%, 14.9%, and 5.5%, respectively). Eggs were laid beneath leaf epidermis, concentrated around leaf veins. Under continuous darkness, total nymphs (284) did not differ from photoperiod (L:D = 18:6) (308). The numbers of stem and pod-hatched nymphs were decreased, while the leaf-hatched nymphs increased significantly (46 to 106). In conclusion, *M. usitatus* prefers pods for oviposition. Darkness does not alter total reproductive output but increases oviposition and hatch rate in leaves, shifting egg spatial distribution among plant parts.

## Simple Summary

Bean flower thrips are tiny insects that damage cowpea, an important vegetable crop in Asia. This study investigated which parts of the cowpea plant (stems, leaves or pods) the females prefer to lay their eggs on, and whether darkness would alter their choices. Histological observation revealed that the female thrips insert their eggs beneath the plant epidermis. The oviposition depth was the shallowest on stems, intermediate on leaves, and the deepest in pods. Under the natural light condition (L:D = 18:6), females preferred to lay most of their eggs inside pods, where the eggs also hatched best. They laid fewer eggs on leaves and very few on stems. When we kept the insects in continuous darkness, the total number of eggs and hatched nymphs did not change significantly. However, females laid more eggs on leaves, and the hatched nymphs on leaves were more than those under the normal light condition. The eggs took 4–7 days to hatch. These findings show that bean flower thrips can adjust their egg-laying behavior under different light conditions, which might help us to understand the distribution of eggs and nymphs in cowpea plants of different light conditions, so as to control the reproduction of thrips.

## 1. Introduction

Thrips (Thysanoptera: Thripidae), mainly distributed in subtropical or tropical regions, are hemimetabolous insects with a life cycle comprising egg, nymph, prepupa, and adult stages [1]. Thrips feed on plant cell contents via their unique rasping-piercing-sucking mouthparts. They scrape and lacerate leaf surface cells to release sap for ingestion, which creates distinct scarring damage on host plants. Beyond this direct physical injury, thrips serve as major transmission vectors for a wide range of plant pathogenic viruses [2,3,4]. Except some kinds of thrips, such as suborder Tubulifera, which includes the single family Phlaeothripidae (e.g., *S. linguis*), which lay eggs only on plant surfaces, most females insert their eggs into plant tissues using a saw-like ovipositor, thereby injuring the plant [5]. Consequently, both feeding and oviposition by thrips cause tissue damage throughout the crop growing season, decreasing the commercial value of agricultural products [4,6].

*Megalurothrips usitatus* (Thysanoptera: Thripidae), widely distributed in Asia, is one of the major pests of cowpea and other leguminous crops and has caused significant agricultural economic losses [4,6,7]. In recent years, cowpea yield losses caused by *M. usitatus* in Hainan, China, have reached as high as 70% [8]. It is small, highly cryptic, and has a strong reproductive capacity, enabling it to rapidly form high-density populations under favorable environment, posing a continuous threat to crop production [7]. Like oviposition characteristic of most thrips, the eggs of *M. usitatus* are also inserted into plant tissues [1].

Many insects exhibit behavioral selectivity when ovipositing on different plant species or different parts of the same plant, which is considered a key decision affecting individual fitness [9]. After comparing oviposition by thrips on cotton, soybean and peanut seedlings, Giovani et al. [10] found significant preferences for two host species as follows: the highest number of eggs was laid on the leaves of peanut, while the highest number of nymphs occurred on cotton. *Thrips tabaci* prefers to oviposit at the base of onion leaves, indicating that oviposition intensity varies in different parts of the same leaf [11]. These reports demonstrate that thrips are selective in choosing oviposition sites. However, whether *M. usitatus* exhibit selectivity between different plant parts of the same crop, such as stems, leaves, flowers or fruits, is currently unknown. In addition, does the hatching rate of eggs have plant parts’ specificity? It is still unclear whether insects select specific plant parts or specific sites of the same plant parts based on their ease of insertion, or choose plant tissues with better nutrition.

Photoperiod is another important environmental factor regulating insect behavior, significantly affecting visual responses, phototaxis, and spatial distribution [12,13,14]. It has been reported that different wavelengths and light intensities can alter thrips behavior as follows: different spectral combinations significantly influence the visual response and avoidance behavior of *Frankliniella occidentalis* (Thysanoptera: Thripidae) [15]. Photoperiod can also affect insect reproduction. Chaisuekul and Riley [16] reported that different photoperiods (e.g., 6:18, 12:12, and 18:6) significantly influence the number of eggs laid by *F. occidentalis* and *Thrips tabaci*, with longer light periods resulting in higher fecundity.

Although the effects of light on thrips behavior and reproduction from the perspectives of spectral characteristics and photoperiod has been explored, most have focused on phototaxis or overall fecundity. How photoperiod changes affect the distribution of eggs in different host tissues remains unclear. In particular, for *M. usitatus*, it is also uncovered whether changing photoperiod conditions will alter the distribution of eggs among stems, leaves or pods.

In response to the two issues, we hypothesized that *M. usitatus* has a tendency to lay eggs in different plant parts of cowpea, and that different photoperiods affect egg laying and hatching rate of nymphs. Therefore, this study compared the number of eggs laid and nymphs hatched by *M. usitatus* in different cowpea plant parts (stems, leaves, and pods) under photoperiod (L:D = 18:6) and continuous darkness to analyze the effects and reasons of photoperiod and host plant part type on oviposition choice. The histological oviposition site of cowpea plant parts (stems, leaves, and pods) was observed too. The results will help elucidate the regulatory role of photoperiod on oviposition behavior and reveal the distribution characteristics of eggs among different host plant parts.

## 2. Materials and Methods

### 2.1. Insect

*Megalurothrips usitatus* were collected from a cowpea experimental field of the Institute of Plant Protection, Hainan Province, China, and subsequently reared under laboratory conditions in College of Plant Protection, China Agricultural University, Beijing, China.

Adult *M. usitatus* (20 males and 20 females mixed) were reared in Petri dishes, which were kept in artificial climate incubators (Ningbo Jiangnan Instrument Factory, Ningbo, Zhejiang, China), provided fresh kidney bean pods as food every two days and maintained at 26 ± 1 °C, relative humidity (RH) of 70–75% and a photoperiod (L:D = 18:6).

### 2.2. Plant Material

The cowpea (cultivar “Fengchan 168”, Beijing, China) was grown in an artificial climate chamber under conditions of temperature 22 ± 1 °C and photoperiod (L:D = 18:6). Plants were grown in 48 black plastic pots (diameter 14 cm, height 7 cm) filled with potting soil, one plant per pot, and watered once a week without fertilizers or pesticides.

After 20–25 days, cowpea seedlings with one pair of true leaves were selected for experiments. Mature cowpea pods of the same cultivar were purchased from a local market (supplied by Xianghe Fenglian Fruit and Vegetable Planting Cooperative, Langfang, Hebei, China).

All seedlings and pods used in the assay were screened to maintain consistent size and developmental stage.

### 2.3. Behavioral Experiments

**Figure 1:**
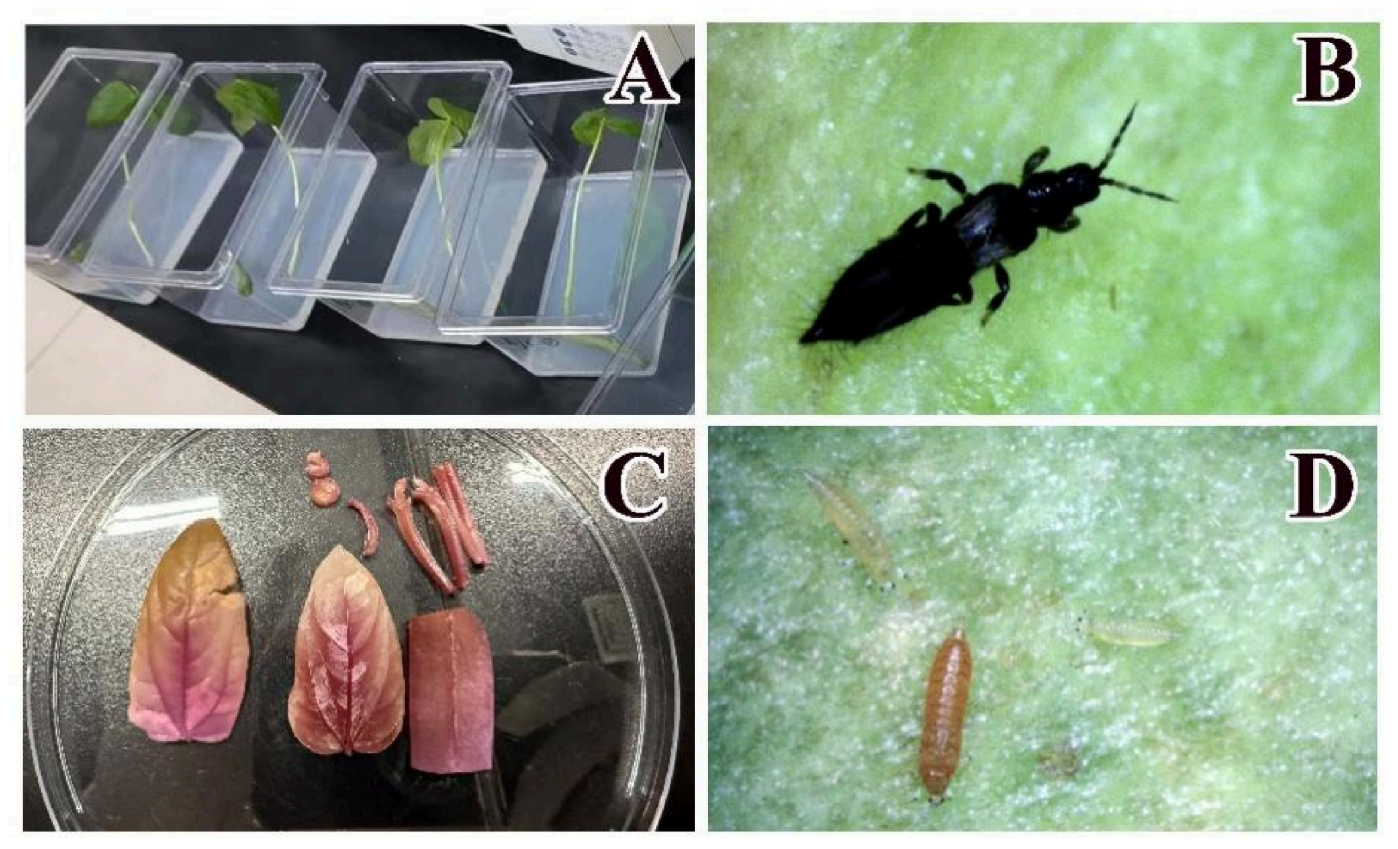
(**A**) Rearing box with a 3–5 mm layer of agar medium at the bottom, containing 10 mated female adults of *M. usitatus*, one intact 20–25-day-old cowpea seedling and one pod segment. (**B**) Mated female adults of *M. usitatus*. (**C**) Stained cowpea plant parts for egg counting with basic fuchsin. (**D**) Nymphs of *M. usitatus* hatched from eggs laid on cowpea.

Firstly, 3–5 cm thick layer of agar medium (1.2 g/100 mL) was placed in the insect rearing boxes (20 × 10 × 14 cm^3^); a cowpea seedling (20–25 days old, two pairs of true leaves) was cut at the soil surface with alcohol-disinfected scissors (oblique cut) and then inserted obliquely into the agar to prevent wilting. Cowpea pods were cut to a length 5 cm shorter than the stem and also placed obliquely in the agar. Each box received one stem with two compound leaves and one pod (Figure 1A). Ten mated female *M. usitatus* (Figure 1B) were introduced into each box. After inoculation, the opening of each rearing box was sealed with plastic wrap to stop thrips from escaping. Twenty pinholes were pricked through the wrap with an insect needle for ventilation, and parafilm was wrapped around the edge for extra sealing.

Twenty-four insect rearing boxes were used in the experiment, divided into the following two groups equally (12 in each group): (1) photoperiod (L:D = 18:6) group (12 boxes): the boxes were placed in an incubator at 26 ± 1 °C, 70–75% RH, L:D = 18:6. (2) Continuous darkness (L:D = 0:24) group (12 boxes): the same procedure was followed, but boxes were kept in continuous darkness.

After 24 h of incubation, the cowpea stems, leaves and pods were collected from each group (6 boxes), stained with basic fuchsin (Figure 1C), and the number of eggs was counted under a stereomicroscope (Chongqing Optec Instrument Co., Ltd., Chongqing, China). In the remaining insect rearing boxes (6 boxes), the pod and seedling were removed too. Leaves were cut from the stem, and roots were removed. The segments of pods, leaves and stem in the same box were labeled and placed separately into Petri dishes containing 1–2 cm of agar, and incubated under the same conditions for 7 days. The number of hatched nymphs (Figure 1D) was recorded daily in each Petri dish. This procedure was performed simultaneously for both the photoperiod (L:D = 18:6) group and the continuous darkness group.

An additional 6 cowpea seedlings were used, and one pair of leaves was taken from each seedling. The leaves were placed in Petri dishes with a 1 cm layer of agar (1.2%): 6 leaves with adaxial side up and 6 with abaxial side up (one leaf of each pair adaxial-up, the other abaxial-up). Five female *M. usitatus* were randomly introduced to each leaf. Dishes were incubated at 26 ± 1 °C, 70–75% RH, under either L:D = 18:6 or continuous darkness for 24 h. After 24 h, the leaves were removed, stained with basic fuchsin for eggs counting.

### 2.4. Eggs Counting

The outermost tissue layer (approximately 5 mm thick) of cowpea stems and pods was excised with a scalpel blade. The excised tissues, together with leaf samples, were stained in staining solution (0.2% basic fuchsin prepared in a 1:1 mixture of 70% ethanol and glacial acetic acid) for 24 h. Following staining, all samples were gently rinsed three times with distilled water and subsequently fixed in Fixing Solution (distilled water: 99% glycerol: 85% lactic acid = 1:1:1) for 48 h. The treated stems, pods and leaves were laid flat in a distilled water-filled Petri dish, and egg numbers were quantified under a stereomicroscope.

### 2.5. Scanning Electron Microscopy

Leaf samples collected from the above experiment were fixed in 75% ethanol and subjected to serial ethanol dehydration. Target regions containing eggs were mounted on specimen stubs, air-dried, and sputter-coated with gold. Samples were observed under a scanning electron microscope (TM4000, Hitachi High Tech, Tokyo, Japan) via secondary electron imaging at an accelerating voltage of 10 kV, emission current of 10 μA and probe current of 13 nA, and micrographs were captured.

### 2.6. Histological Staining

Cowpea pods, leaves and stems containing eggs (after 24 h of incubation under photoperiod (L:D = 18:6)) were fixed in 10% formalin overnight. Samples were then dehydrated through graded ethanol and xylene, embedded in paraffin, and sectioned at 6 μm thickness. Routine hematoxylin and eosin (HE) staining was performed, and sections were observed.

### 2.7. Data Analysis

Data were analyzed using SPSS statistical software 16.0 (IBM Corp., Armonk, NY, USA).

The Shapiro–Wilk test (for small sample) was performed to assess normality and homogeneity of variance tests was used to evaluate variance homogeneity. Normal distribution data were analyzed using one-way analysis of variance (ANOVA) or independent sample *t*-test, while non-normal distribution data were tested using nonparametric test; Welch test and corrected *t*-test were used for the data with heterogeneous variance.

Therefore, ANOVA was used to compare the number of eggs in different cowpea plant parts under L:D = 18:6 photoperiod, the number of eggs on different cowpea plant parts under continuous darkness, and the shortest distance between eggs and plant surface in different cowpea plant parts. Welch test was used to compare the number of nymphs on different cowpea plant parts under different photoperiods. Independent-sample *t*-tests were used to compare the numbers on the adaxial and abaxial surfaces of cowpea leaves, the number of eggs on different cowpea plant parts under different photoperiods, and hatched nymph numbers between different photoperiods in the same plant parts. Mann–Whitney test (nonparametric test) was used to compare cumulative number and daily number (1–7 days) of hatched nymphs under different photoperiods. The significance level was set at *p* < 0.05.

## 3. Result

### 3.1. Comparison of Oviposition Choice in Different Cowpea Plant Parts Under Photoperiod (L:D = 18:6)

**Figure 2:**
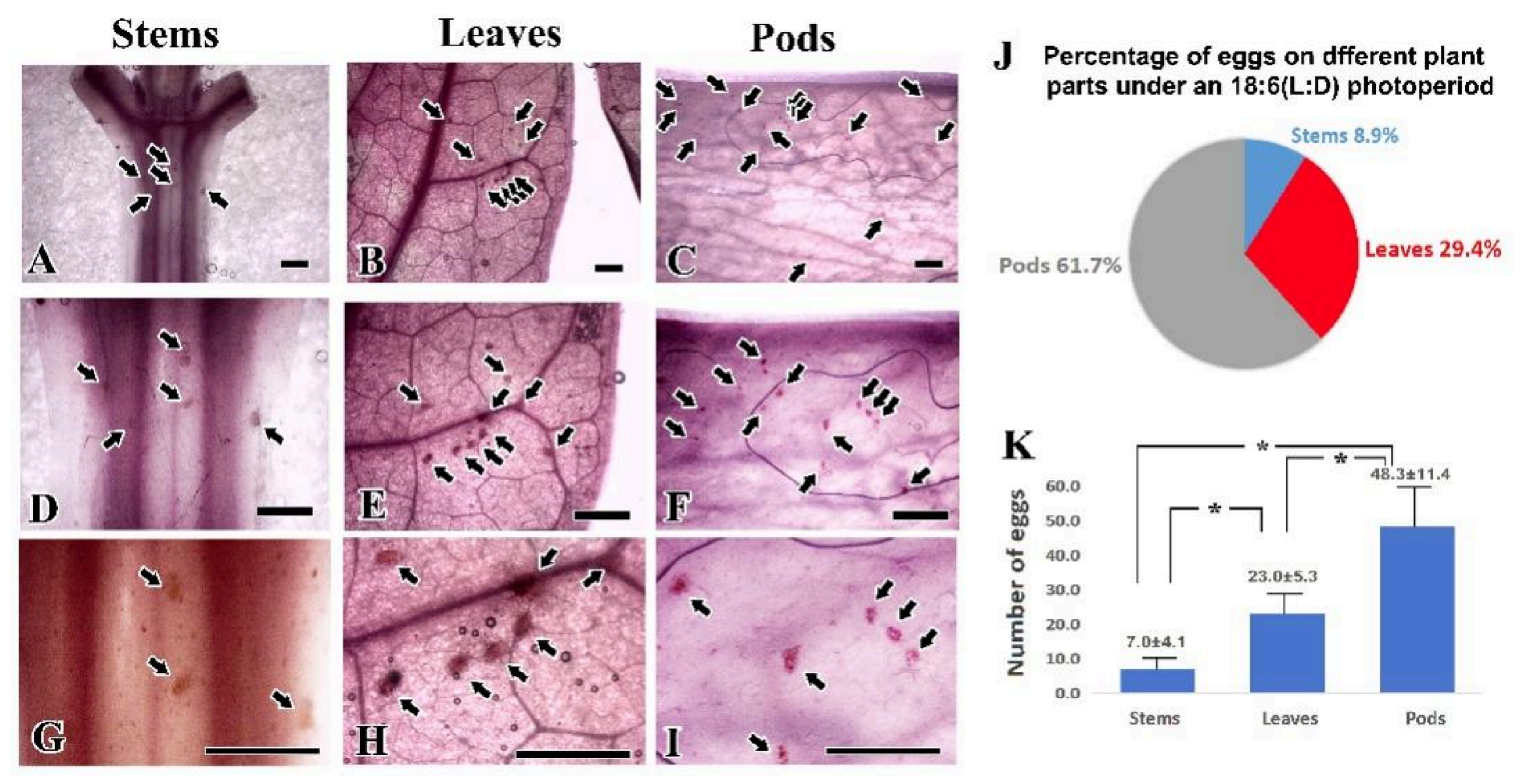
*M. usitatus* eggs distribution in cowpea stems, leaves and pods under photoperiod (L:D = 18:6). (**A**–**I**) *M. usitatus* eggs (indicated by arrows) within stems (**A**,**D**,**G**) leaves (**B**,**E**,**H**) and pods (**C**,**F**,**I**) tissues under a stereomicroscope. (**J**) Pie chart illustrating the percentage distribution of *M. usitatus* eggs on different cowpea plant parts. (**K**) The number of *M. usitatus* eggs deposited on different cowpea plant parts (*n* = 6; α = 0.05; ANOVA). Values are mean ± SD (Standard Deviation). For multiple comparisons, “*” denotes significant difference in stems, leaves and pods according to Least Significant Difference (LSD) test. Scale bar = 500 μm.

*Megalurothrips usitatus* eggs stained with staining solution in cowpea stems, leaves and pods were observed and counted (Figure 2A–I). Under photoperiod (L:D = 18:6), the total number of eggs in the six boxes was 470 (mean 78.3 per box), and the numbers of eggs on stems, leaves and pods were 42, 138, and 290, respectively (mean 7.0, 23.0, and 48.3 per box), accounting for 8.9%, 29.4% and 61.7% (Table 1; Figure 2J). The differences among the three plant parts were statistically significant (F = 45.03, *p* < 0.001; Figure 2K), which suggested that *M. usitatus* has a clear oviposition preference among different plant parts.

**Figure 3:**
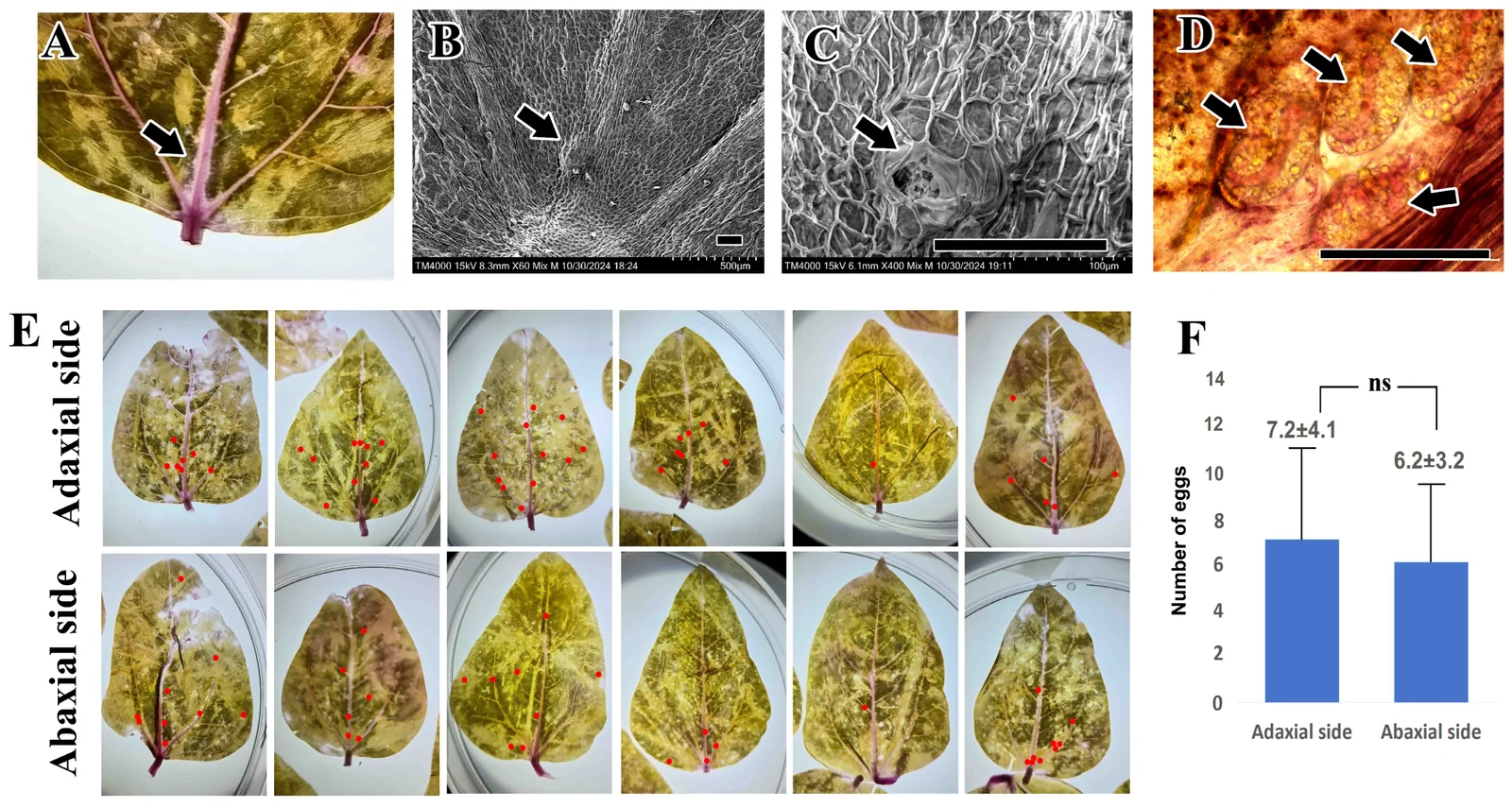
Distribution of *M. usitatus* eggs on cowpea leaf tissues. (**A**–**D**) *M. usitatus* eggs indicated by the arrows in cowpea leaf tissues under stereomicroscope (**A**), scanning electron microscope (**B**,**C**) and optical microscope (**D**). (**E**) Schematic diagram illustrating the distribution of *M. usitatus* eggs on cowpea leaves. (**F**) The number of *M. usitatus* eggs on the adaxial and abaxial surfaces of cowpea leaves (*n* = 6; α = 0.05; *t* test). “ns” denotes no significant difference. Scale bar = 150 µm.

Upon closed examination of oviposition sites on plant parts, no specific distribution pattern was found in the stems or pods. However, in the leaves, most eggs were distributed around leaf veins (Figure 3A–E), indicating that *M. usitatus* has a positional preference when ovipositing inside cowpea leaves.

We also observed 12 leaves (six adaxial-up and six abaxial-up) (Figure 3E), and found that the mean number of eggs in adaxial-up leaves was 7.2, and in abaxial-up leaves it was 6.2, with no significant difference (Figure 3F, *t* = 0.47, *p* = 0.648, *t* test), which indicated that *M. usitatus* has no preference of oviposition behavior towards the adaxial or abaxial side of the leaves.

### 3.2. Number of Hatched Nymphs of M. usitatus on Different Cowpea Plant Parts Under Photoperiod (L:D = 18:6)

**Figure 4:**
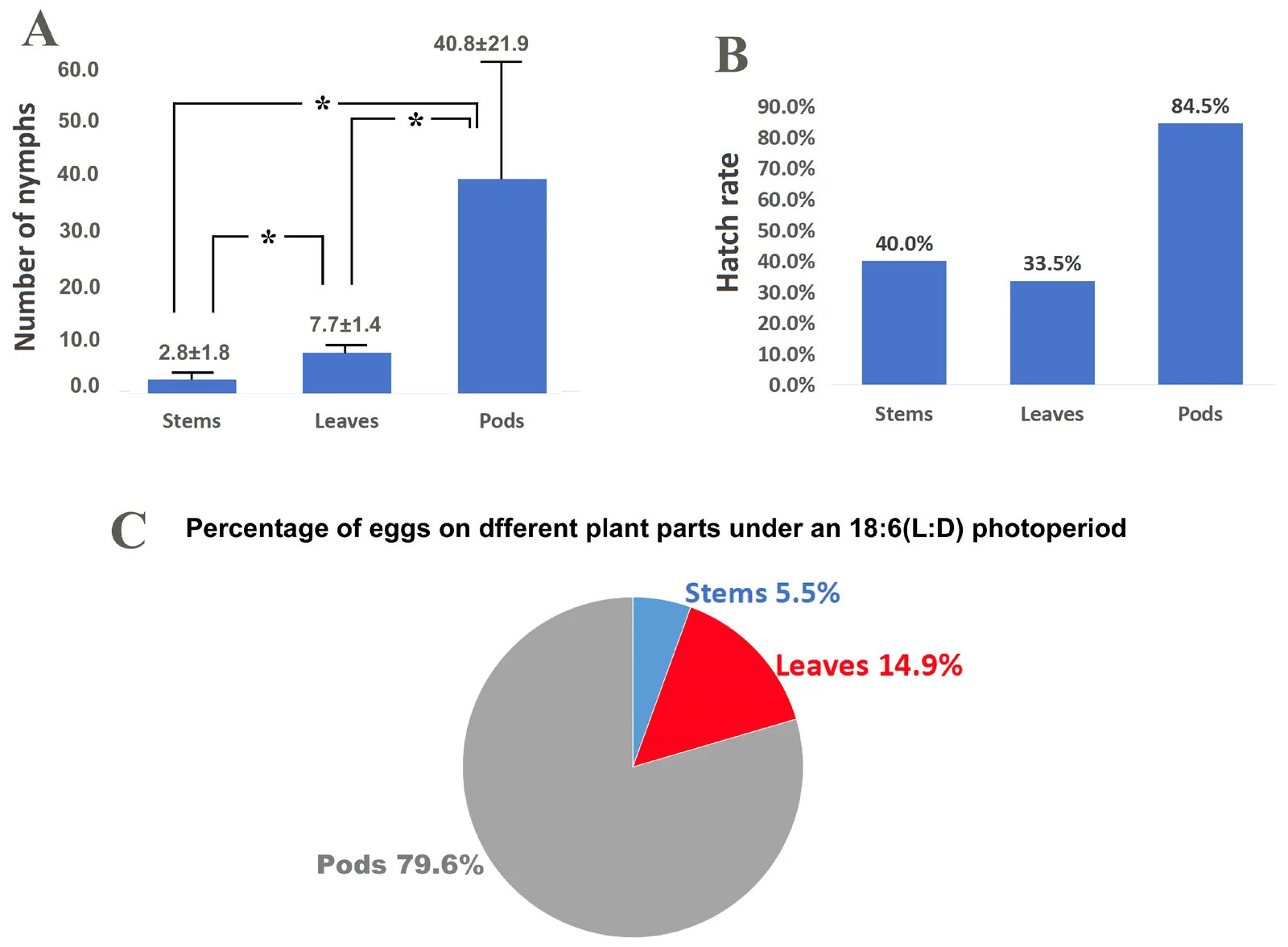
*M. usitatus* nymphs on different cowpea plant parts under photoperiod (L:D = 18:6). (**A**) The number of *M. usitatus* nymphs on different cowpea plant parts (*n* = 6; α = 0.05; Welch test). Values are mean ± SD (standard deviation). For multiple comparisons, “*” denotes significant difference in stems, leaves and pods according to Games–Howell test. (**B**) The hatching rate of *M. usitatus* on different cowpea plant parts. (**C**) The percentage proportion of *M. usitatus* eggs on different cowpea plant parts.

The total number of hatched nymphs in the six culture boxes was 308 (mean 51.3 per box), and the numbers of nymphs on stems, leaves and pods were 17, 46, and 245 (mean 2.8, 7.7, 40.8 per box), accounting for 5.5%, 14.9%, and 79.5%, with significant differences (Table 1; Figure 4A,C). The differences among the three plant parts were statistically significant (Figure 4A, F = 19.46, *p* = 0.001, Welch test). The hatching rate was calculated as the number of *M. usitatus* nymphs divided by the number of deposited *M. usitatus* eggs. The overall hatch rate was 68.7%. Hatch rates on stems, leaves and pods were 40.0%, 33.5%, and 84.5%, respectively (Figure 4B). These results indicated that different plant parts affected egg hatching success, with pods being most favorable, suggesting that *M. usitatus* could recognize pods as the place where hatching success is higher.

### 3.3. Histological Comparison of in Different Cowpea Plant Parts During Oviposition of M. usitatus

**Figure 5:**
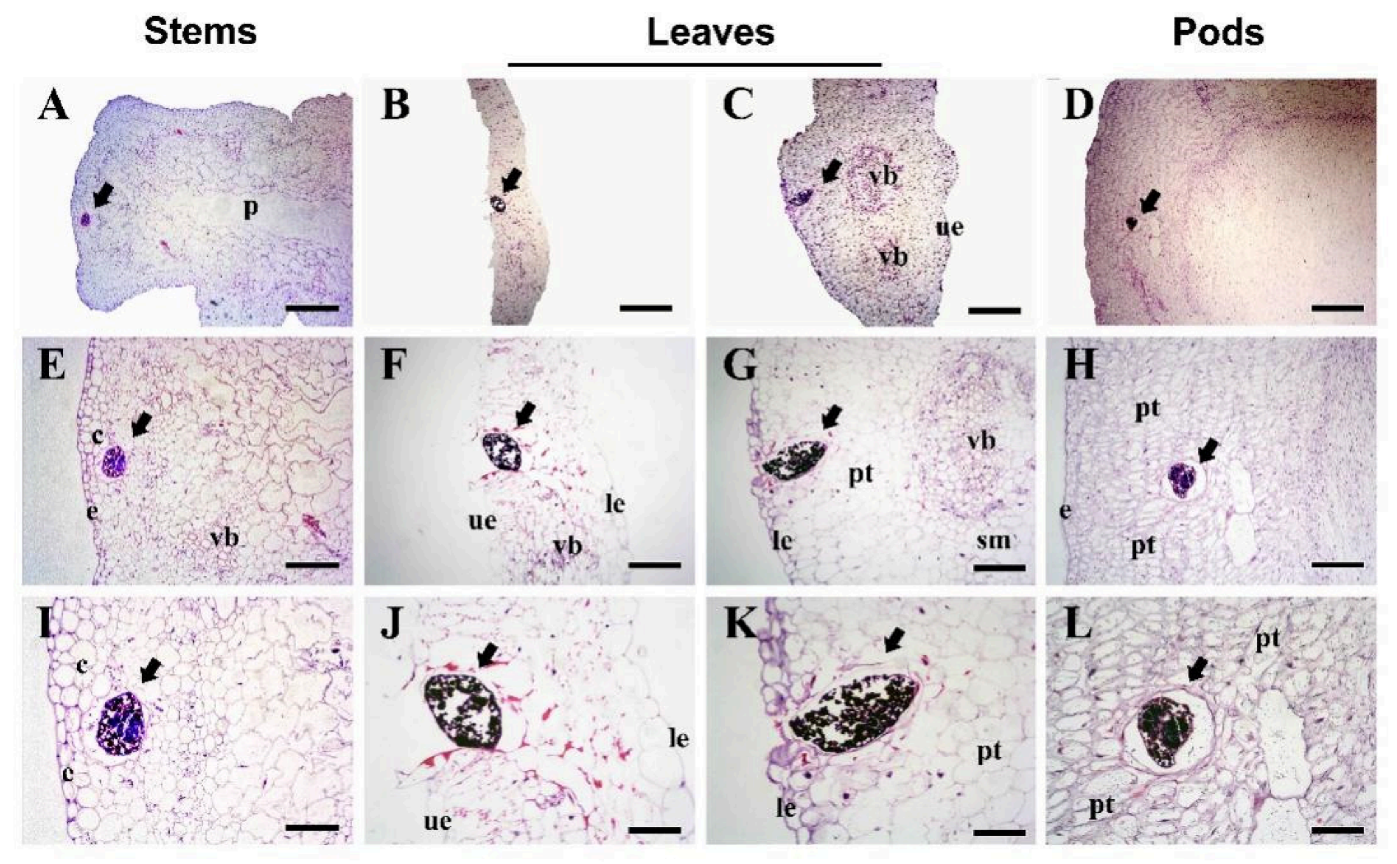
HE staining of oviposition sites of *M. usitatus* in cowpea stems, leaves and pods under photoperiod (L:D = 18:6). Micrographs of *M. usitatus* eggs within cowpea stems (**A**,**E**,**I**), leaves (**B**,**C**,**F**,**G**,**J**,**K**) and pods (**D**,**H**,**L**). p: pith, vb: vascular bundle, ue: upper epidermis, le: lower epidermis, pt: parenchymal tissue, sm: spongy mesophyll, c: cortex, e: epidermis. Scale bar: (**A**–**D**) = 250 μm; (**E**–**H**) = 100 μm; (**I**–**L**) = 50 μm.

Twenty-four hours after laying eggs, HE staining was performed on the pods, leaves and stems, and the results showed that the eggs were located beneath the plant epidermis. The parenchymal tissue surrounding the egg was abundant in pods and leaves, but sparse in the stem (Figure 5). Measuring the position of eggs in histological sections, it was found that the shortest distance between the egg and the surface of epidermis was 19.1 μm in leaves, followed by 38.2 μm in stems and 202.6 μm in pods, with significant difference (Table 2; F = 584.87, *p* < 0.001, ANOVA). Therefore, histological observations have confirmed that *M. usitatus* lays eggs beneath the epidermis of plant parts, and the cellular environment around the eggs varies depending on the plant parts. The depth of the eggs in different plant parts also varies greatly, which may lead to differences in the hatching of nymphs.

### 3.4. Effects of Photoperiod on Oviposition Preference

After 24 h in continuous darkness, the total number of eggs in six culture boxes was 460 (mean 76.7 per box), which did not differ from the 470 (mean 78.3 per box) eggs recorded under photoperiod (L:D = 18:6).

**Figure 6:**
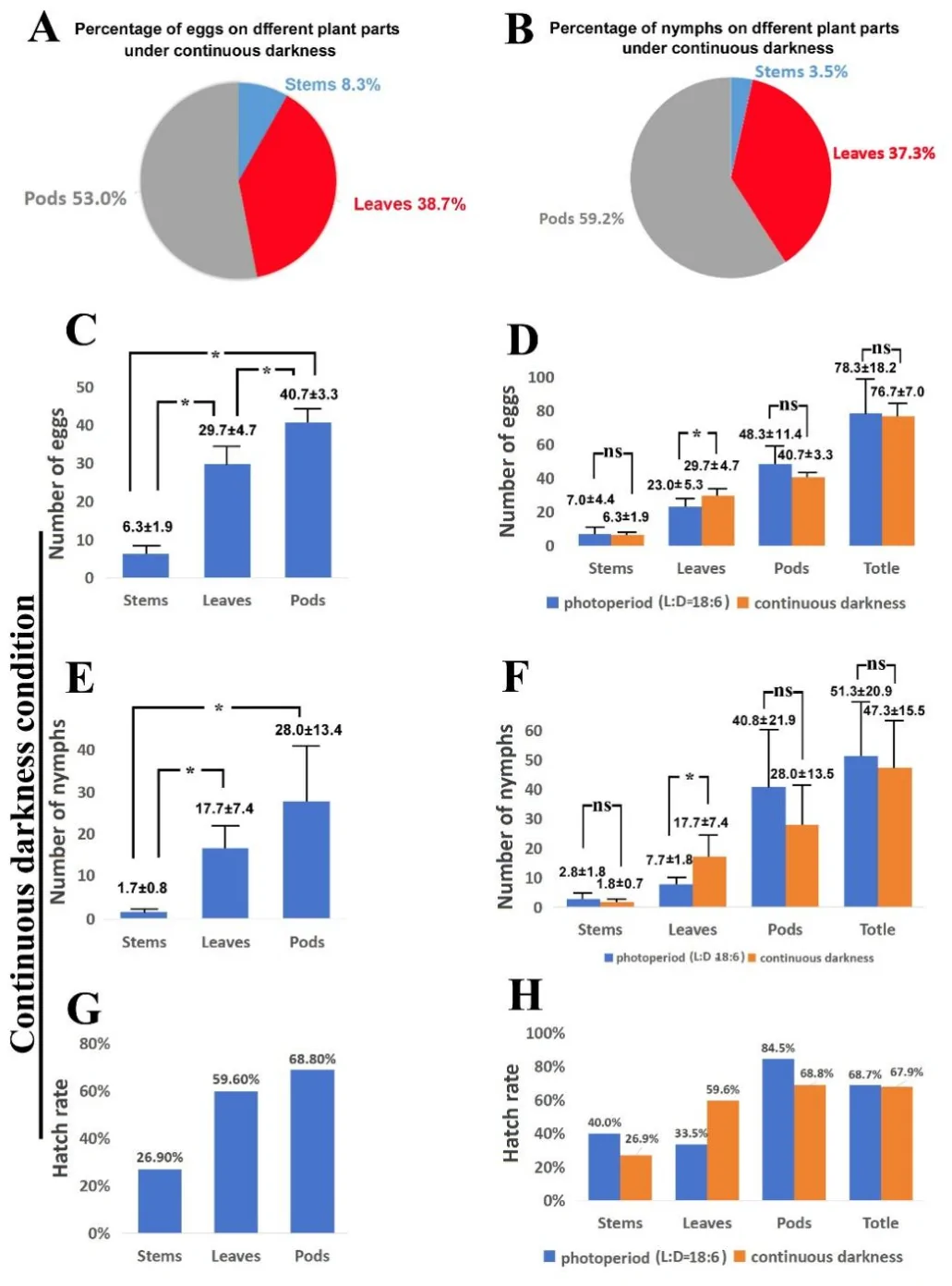
*M. usitatus* eggs deposited and nymphs on different cowpea plant parts under continuous darkness. (**A**) The percentage distribution of *M. usitatus* eggs on different cowpea plant parts under continuous darkness. (**B**) The percentage distribution of *M. usitatus* nymphs on different cowpea plant parts under continuous darkness. (**C**) The number of *M. usitatus* eggs deposited on different cowpea plant parts (*n* = 6; α = 0.05; ANOVA). Values are mean ± SD. For multiple comparisons, “*” denotes significant difference in stems, leaves and pods according to LSD test. (**D**) The number of *M. usitatus* eggs on different cowpea plant parts under photoperiod (L:D = 18:6) and continuous darkness (*n* = 6; α = 0.05; *t* test). “*” denotes significant difference, and “ns” denotes no significant difference. (**E**) The number of *M. usitatus* nymphs on different cowpea plant parts under continuous darkness (*n* = 6; α = 0.05; Welch test). Values are mean ± SD. For multiple comparisons, “*” denotes significant difference in stems, leaves and pods according to Games–Howell test. (**F**) The number of *M. usitatus* nymphs on different cowpea plant parts under photoperiod (L:D = 18:6) and continuous darkness (*n* = 6; α = 0.05; *t* test). “*” denotes significant difference, and “ns” denotes no significant difference. (**G**) The hatching rate of *M. usitatus* on different cowpea plant parts under continuous darkness. (**H**) The hatching rate of *M. usitatus* on different cowpea plant parts under photoperiod (L:D = 18:6) and continuous darkness.

The oviposition preference of plant parts was similar to photoperiod (L:D = 18:6): pods highest, then leaves, and then stems, with egg numbers of 244 (53.0%), 178 (38.7%), and 38 (8.3%), mean 40.7, 29.7, and 6.3 per box respectively, with significant differences among three plant parts (Figure 6C; F = 153.69, *p* < 0.001, ANOVA). Compared with photoperiod (L:D = 18:6), the eggs’ number of leaves were statistically increased (Figure 6D: *t* = 2.31, *p* = 0.043, *t* test). However, there was no statistical significance of egg number in stem, pods and sum between photoperiod (L:D = 18:6) and continuous darkness (stem: *t* = 0.37, *p* = 0.722; pods: *t* = 1.59, *p* = 0.143; sum: *t* = 0.21, *p* = 0.839, *t* test).

After 7 days of incubation, the total number of hatched nymphs under darkness was 284 (mean 47.3 per box), which did not differ significantly from the 308 nymphs under photoperiod (L:D = 18:6) (Table 1). The numbers of nymphs were still highest on pods, followed by leaves and stems: 168, 106, and 10 (mean 28.0, 17.7, 1.7 per box), respectively (59.2%, 37.3%,3.5%). Nymph numbers in leaves and pods were significantly higher than in stems (Figure 6E: F = 22.83, *p* = 0.001, Welch test; stems vs. leaves, *p* = 0.009, Games–Howell test; stems vs. pods, *p* = 0.014, Games–Howell test), but the difference between leaves and pods was not significant (Figure 6E: *p* = 0.362, Games–Howell test). The overall hatch rate under darkness was 67.9%, similar to photoperiod (L:D = 18:6) (68.7%) (Table 1; Figure 6H).

Further analysis revealed that continuous darkness substantially altered the distribution of newly hatched nymphs across cowpea plant parts relative to the 18:6 light-dark photoperiod. Both nymph abundance and hatching rates declined on stems and pods under dark conditions. In contrast, the number of nymphs emerging from leaves under continuous darkness was significantly 2.3-fold higher than that under the L:D = 18:6 regime (Figure 6F: *t* = 3.246, *p* = 0.021, *t* test). Correspondingly, the leaf hatching rate rose sharply to 59.6%, a prominent increase compared with the 33.5% observed under the normal photoperiod (Figure 6G,H). These results indicated that darkness only slightly elevated total egg deposition on leaves, and the striking rise in leaf-derived nymphs was primarily driven by a significant improvement in leaf egg hatching success. We infer that dark environments are conducive to the embryonic development and hatching of thrips eggs laid on leaves.

### 3.5. Daily Hatching Dynamics in Different Cowpea Plant Parts Under Different Photoperiods

**Figure 7:**
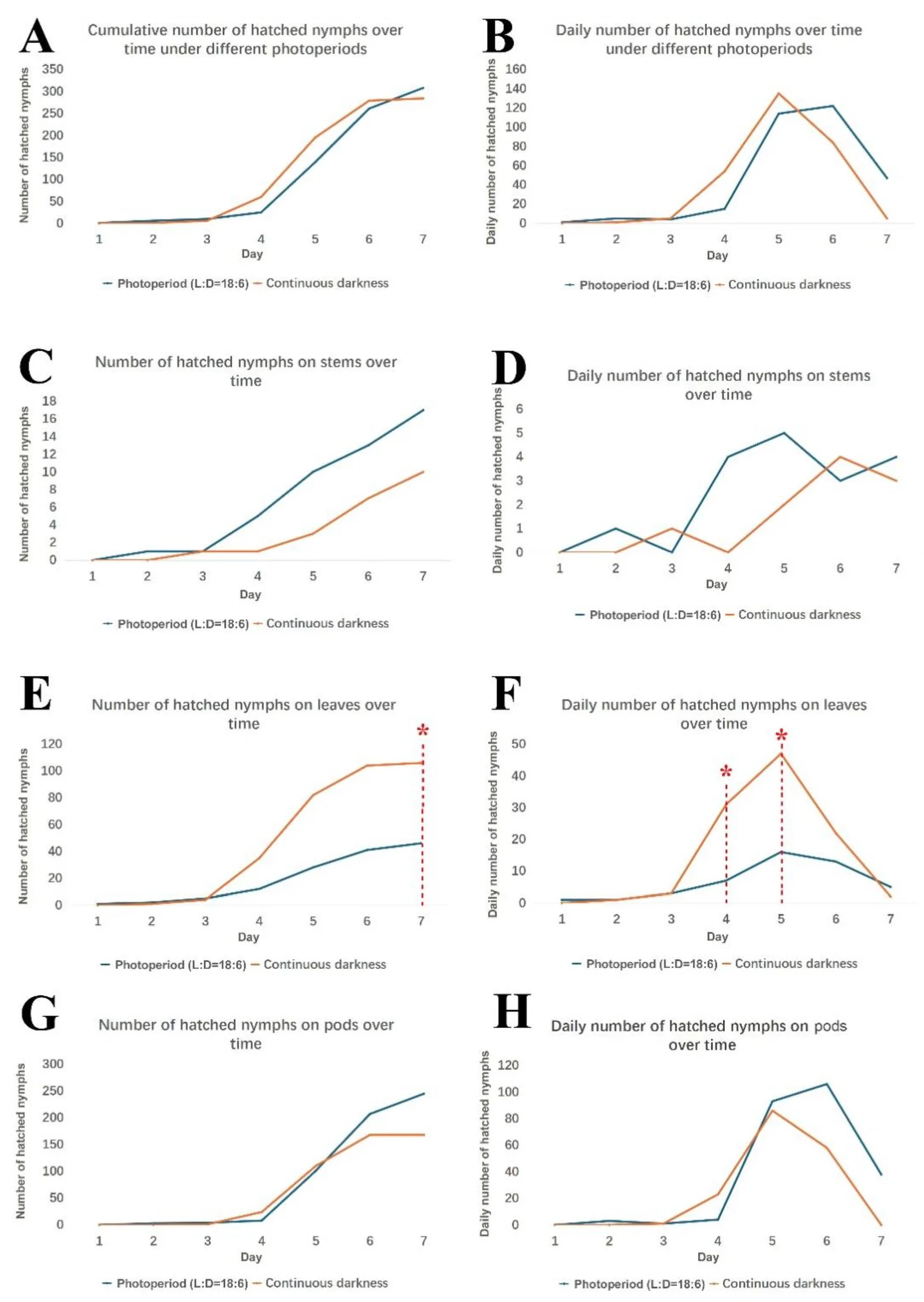
(**A**,**B**): Cumulative number (**A**) and daily number (**B**) of hatched nymphs under continuous darkness and L:D = 18:6 photoperiod. (**C**,**D**): Cumulative number (**C**) and daily number under different photoperiods on stems. (**E**,**F**): Cumulative number (E: *n* = 6; α = 0.05; *t* test) and daily number (F: *n* = 6; α = 0.05, Mann–Whitney test) of hatched nymphs on leaves. “*” denotes significant difference. (**G**,**H**): Cumulative number (**G**) and daily number (**H**) of hatched nymphs on pods.

The cumulative number of hatched nymphs over 7 days under continuous darkness and L:D = 18:6 photoperiod is similar (Figure 7A). Daily counts of nymphs across days after oviposition showed that nymph numbers rose sharply starting on day 4, peaked on day 5 or day 6, and then declined (Figure 7B). On days 4 and 5, significantly more nymphs hatched from leaves under continuous darkness than under the L:D = 18:6 photoperiod (Figure 7F; day 4: Z = 2.185, *p* = 0.029; day 5: Z = 2.093, *p* = 0.036, Mann–Whitney test). The increase in the number of nymphs in the two days eventually produced a significantly higher total number of leaf nymphs in the dark treatment (Figure 6F and Figure 7E: *t* = 3.246, *p* = 0.021, *t* test). No significant differences between the two different photoperiods were detected for the remaining plant parts at any sampling time point (*p* > 0.05). Overall, dark conditions boosted the quantity of hatched nymphs on leaves, and most leaf eggs hatched around days 4 and 5.

## 4. Discussion

We systematically investigated the oviposition preference of *M. usitatus* in different cowpea plant parts and its regulation by photoperiod. The main findings include the following: (1) under photoperiod (L:D = 18:6), the oviposition preference ranked as pods > leaves > stems, with egg percentages of 61.7%, 29.4% and 8.9%, respectively. The percentages of hatched nymphs on pods, leaves and stems (79.5%, 14.9%, and 5.5%) followed a similar trend. However, the hatch rates were not consistent with the oviposition preference: stems 40.0%, leaves 33.5%, and pods 84.5%. (2) Under continuous darkness condition, the total number of eggs laid and the overall hatch rate did not change significantly, but the number of hatched nymphs on leaves increased markedly (from 46 to 106), and the hatch rate on leaves rose from 33.5% to 59.6%, while the number of hatched nymphs on pods decreased (not significantly). This indicates that under experimental conditions using detached cowpea organs, *M. usitatus* can offset the reduction in egg deposition on pods by increasing oviposition on leaves when exposed to the unfavorable continuous dark treatment, so as to maintain a stable total egg output.

### 4.1. Re-Examination of Oviposition Plant Part Preference and the Preference-Performance Hypothesis

*Megalurothrips usitatus* showed a clear plant part preference for oviposition on cowpea. Under both photoperiod (L:D = 18:6) and continuous darkness, females preferentially laid eggs in pods, which had the highest nymphal hatch rate. Histological observations revealed that pod tissues contain more abundant cellular structures (Figure 3D,H,L), likely providing more nutrients, and the eggs are placed deeper (Table 2), which benefits egg protection, hatching, and survival. Additionally, we found that within the same leaf, females exhibited a specific internal preference: they favored laying eggs around the leaf veins, as reported by Hameed et al. [17], but did not distinguish between the upper and lower surfaces of the leaf. This is consistent with the classical oviposition preference-performance hypothesis, which proposes that females should select host sites that maximize offspring survival and development [18].

However, this hypothesis may not always be optimal. In the study, leaves had 29.4% of the eggs, far more than stems (8.9%) under photoperiod (L:D = 18:6), but the hatch rate on leaves was only 33.5%, lower than that on stems (40.0%), which suggested that the “preference” sites (leaves) chosen by female insects did not lead to higher “offspring performance” (hatch rate), but lower performance. From an anatomical perspective, although leaf veins can provide more fluids and nutrients, thinner leaves result in superficial egg laying (Figure 3F,G, Table 2), making them more susceptible to external interference and less protected from plants, thereby reducing the success rate of hatching.

Another possibility is that the oviposition decisions are not solely based on the survival of their offspring, but rather adopt a strategy of “risk diversification” across different plant parts. Notably, under continuous dark conditions, the number and proportion of eggs laid on the leaves significantly increased compared to the photoperiod (L:D = 18:6), from 138 (29.4%) to 178 (38.7%), and the hatch rate on the leaves increased to 59.6%. Moreover, on the 5th day, the hatching number was much higher than that of the photoperiod (L:D = 18:6) (*p* < 0.05). This suggests that environmental stress may induce females to adjust their egg allocation in response to unpredictable risks that pods may face, such as predators or physical damage. The “oviposition site shift strategy”, which might be considered as a supplement to the oviposition preference-performance hypothesis, has rarely been reported in thrips, indicating that oviposition preference should not be regarded as a fixed trait, but may change under environmental influences.

### 4.2. Regulation of Oviposition Behavior by Photoperiod

By comparing the condition of photoperiod (L:D = 18:6) and continuous darkness, continuous darkness did not alter the total number of eggs and nymphs, but significantly changed the distribution of oviposition sites and hatch rates in different plant parts. This phenomenon is not completely consistent with findings on other thrips: *Frankliniella occidentalis* can maintain some reproductive capacity under different photoperiods, while its nymph numbers decrease significantly under continuous darkness [19,20]. The relatively stable nymph numbers observed in *M. usitatus* in our study reflect species-specificity and also reveal that *M. usitatus* might adjust its oviposition strategy to maintain a stable reproductive output when facing environmental stress, demonstrating strong behavioral plasticity under different photoperiods.

The following three mechanisms may explain the issue: (1) reduced visual orientation: *M. usitatus* may rely on visual cues for the location of pods. Under darkness, vision is ineffective, and females may switch to tactile or chemical cues. Leaves, with their large surface area and frequent physical contact with the ovipositor, may become the default substrate. Previous studies have shown that *F. occidentalis* can use olfactory cues to locate plants [21]. (2) Changes in the leaf micro-environment: darkness may alter leaf surface humidity, temperature or volatile emission, making it a more suitable oviposition substrate. The increase in leaf hatch rate from 33.5% to 59.6% under darkness strongly suggests that darkness might improve leaf suitability (e.g., increasing stomatal aperture) or reduce photoperiod-induced inhibition of embryonic development. (3) Physiological rhythms and hormonal regulation: photoperiod may regulate female endocrine hormone levels, thereby modulating ovipositor penetration force or oviposition drive. *Frankliniella occidentalis* possesses a distinct circadian rhythm, with individuals showing higher activity levels under light exposure compared with dark conditions [22].

Daily counts of nymphs over the 7 days after oviposition revealed a clear temporal concentration of hatching as follows: under both photoperiod (L:D = 18:6) and darkness, nymph numbers began to increase sharply on day 4 and declined after day 7. This dynamic matches the embryonic development period of *M. usitatus*, which is typically 4–6 days under suitable conditions [5,23], indicating that the hatching peak coincides with the egg maturation period. It also suggests that changes in nymph distribution are mainly due to adjustments in adult oviposition behavior rather than a direct effect of photoperiod on the hatching process.

### 4.3. Histological Observation

HE staining showed that the parenchymal tissue surrounding eggs was abundant in pods and leaves, but sparse in stems. Abundant parenchymal tissue may provide better nutrition or mechanical support, favoring egg development and hatching. Histological observation also indicated that the eggs were positioned much deeper in pods than in stems and leaves (Table 2, Figure 3D,H,L). Such deeper oviposition sites may provide greater structural support and more abundant nutrients, which could explain the preference of thrips for ovipositing in pods and the higher hatching rate of eggs within pod tissues.

In addition, eggs on leaves were concentrated around leaf veins, while no obvious positional preference was observed on stems or pods. The reason may be related to the following: (1) the leaf vein area is rich in vascular bundles, which can provide more water and nutrients; (2) plant growth may squeeze the eggs [10]. In contrast, the plant tissue around the leaf veins has fully matured and ceases further growth, making it suitable for the survival and development of eggs.

### 4.4. Ecological and Evolutionary Significance

Photoperiodic conditions alter oviposition site selection of *M usitatus*. Compared with photoperiod (L:D = 18:6), the egg numbers were statistically increased in leaves, decreased in stems and pods under continuous darkness, but overall reproductive output (total egg number) was stable. Wild populations of *M usitatus* are commonly exposed to prolonged rainy weather and shaded dark microhabitats formed by barriers. Based on the results, we infer that field populations may shift oviposition from stems and pods to leaves under prolonged dark conditions, thereby maintaining stable total egg quantity and hatching number.

Therefore, we propose that *M. usitatus* might have a backup regulatory system for oviposition: females favor high-quality pod substrates under normal conditions, and switch to leaves as an alternative oviposition strategy when stressed under darkness.

### 4.5. Practical Implications

The results of this study have several practical applications:

(1) On cowpea seedlings (without pods), *M. usitatus* prefers to oviposit on leaves rather than stems. Therefore, pest monitoring and control measures for seedlings should focus on leaves, and insecticides should be applied precisely to the leaves. (2) On mature cowpea plants with pods, *M. usitatus* shows a strong preference for ovipositing on pods. Indicating that at the podding stage, the focus of insecticide application should shift from leaves to pods to better control thrips populations and protect cowpea pod yield. (3) In field management, monitoring or control strategies should not be based solely on oviposition preferences observed under normal photoperiod conditions. Under shade, rainy, or night conditions, the oviposition distribution of *M. usitatus* may change significantly, thereby affecting the spatial distribution of field populations and the dynamics of nymph emergence. Therefore, when developing Integrated Pest Management strategies, the effects of microenvironmental factors such as photoperiod should be taken into account.

Most cowpea seedlings in the field remain intact without damage, whereas the cowpea plant parts used in our laboratory bioassays were excised. Mechanical wounding of cowpea tissues induces shifts in endogenous plant hormones and the biosynthesis of anti- herbivore compounds such as flavonoids [24,25]. Additionally, excised plant tissues undergo changes in texture hardness and volatile profiles [26]. Although many studies have used detached plant tissues for insect behavioral research [27,28,29], there must be certain physicochemical differences between cut plant tissues used in laboratory experiments and undamaged intact tissues grown under field conditions, and such differences may alter the oviposition behavior and egg survival rate of *M. usitatus*. Accordingly, the laboratory findings cannot be directly extrapolated to field ecosystems, and complementary field trials are needed to verify the results of the experiment herein.

## 5. Conclusions

In summary, *M. usitatus* exhibits a clear plant parts preference for oviposition on cowpea, with pods being the most preferred site, followed by leaves, and stems the least preferred. Photoperiod can modulate the preference as follows: under continuous darkness conditions, although the total reproductive output does not change significantly, the plant part preference is adjusted—the number of eggs laid and the hatch rate on leaves increase significantly, and while pods still receive the highest number of nymphs, the difference from leaves is no longer significant. Under dark conditions, females reduce oviposition on pods and increase oviposition on leaves, thereby maintaining overall reproductive output. This means that farmers still need to guard against outbreaks of *M. usitatus* populations even under complete darkness.

Compared with laboratory experimental setups, actual field environments are far more diverse and complex. In the field, light conditions fluctuate constantly, including variable light intensity throughout the day. Field-grown cowpea plants are intact, with natural soil substrates and co-occurring organisms. Given these prominent discrepancies between laboratory and field environments, the findings of the present study cannot be directly extrapolated to practical field management, and supplementary field trials are therefore necessary to corroborate our experimental outcomes.

Furthermore, subsequent research is required to clarify the molecular and physiological pathways mediating the photoperiod response in *M. usitatus*, particularly the signal perception process and downstream regulatory pathways that alter oviposition decisions.

## Figures and Tables

**Table 1:** Total egg and nymphal.

		Stems	Leaves	Pods	Total
photoperiod (L:D = 18:6)	Egg	42 (8.9%)	138 (29.4%)	290 (61.7%)	470 (100%)
	nymph	17 (5.5%)	46 (14.9%)	245 (79.5%)	308 (100%)
continuous darkness	Egg	38 (8.3%)	178 (38.7%)	244 (53.0%)	460 (100%)
	nymph	10 (3.5%)	106 (37.3%)	168 (59.2%)	284 (100%)

Numbers of *M. usitatus* in different cowpea plant parts under a photoperiod (L:D = 18:6) and continuous darkness (*n* = 6).

**Table 2:** Histological comparison of the shortest distance between eggs and plant surface in different cowpea plant parts (*n* = 5).

	Stems (μm)	Leaves (μm)	Pods (μm)
Mean	38.2	19.1	202.6
SD	6.2	5.6	13.8
F	584.872		
*p*	<0.001		

One-way analysis of variance (ANOVA): significant difference in the distances in different cowpea plant parts (*p* < 0.05); For multiple comparisons, all pairwise comparisons among stems, leaves and pods showed significant differences based on the Least Significant Difference (LSD) test.

## Data Availability

The data presented in this study are available on request from the corresponding author.
